# Chronic viral infections and their role in shaping the tumor immune microenvironment

**DOI:** 10.3389/fimmu.2026.1810902

**Published:** 2026-03-16

**Authors:** Huizi Li, Xiulin Jiang, Quanan Zhang, Yihang Yuan

**Affiliations:** 1 Department of Oncology, The Affiliated Jiangning Hospital with Nanjing Medical University, Nanjing, Jiangsu, China; 2 College of Life Science, University of Chinese Academy of Sciences, Beijing, China

**Keywords:** antiviral therapies, cancer, CD8+ T cell exhaustion, chronic viral infection, exosomes, immune evasion, immunosuppression, oncogenic viruses

## Abstract

Chronic viral infections, such as HBV, HCV, EBV, and HPV, contribute to tumorigenesis not only through direct oncogenic effects but also by reshaping the tumor immune microenvironment (TIME) via complex immunoregulatory mechanisms. These infections enhance immune suppression and promote metastasis. Viruses induce the accumulation of regulatory T cells (Tregs), myeloid-derived suppressor cells (MDSCs), and immunosuppressive cytokines, while driving CD8^+^ T cell exhaustion and impairing NK cell function, creating an immune environment favorable for tumor survival. Chronic inflammation, pro-angiogenic factors, and signals mediated by exosomes and microvesicles further remodel local and distant microenvironments, forming a “pre-metastatic niche” that supports tumor cell colonization and metastasis. Key signaling pathways, including NF-κB, STAT3, PD-1/PD-L1, and TGF-β, are persistently activated by viral proteins such as HBx and LMP1, reinforcing immunosuppression and metastasis. Based on these mechanisms, combined strategies of antiviral therapy with immune checkpoint inhibitors (ICIs) or targeting exosomes and immunosuppressive pathways show potential to enhance antitumor immunity and limit metastasis. A deeper understanding of the virus-immune-metastasis axis and related biomarkers may provide precise immunotherapeutic strategies for virus-associated cancers and improve patient outcomes.

## 1 Introduction

Chronic viral infections are a major global public health concern and significant risk factor for multiple cancers (1). Persistent infections with viruses such as hepatitis B virus (HBV), hepatitis C virus (HCV), Epstein-Barr virus (EBV), and human papillomavirus (HPV) are strongly linked to specific cancers (2). For example, HBV and HCV are closely associated with hepatocellular carcinoma (HCC), EBV is linked to nasopharyngeal carcinoma and certain lymphomas, and high-risk HPV types are major drivers of cervical and other reproductive tract cancers (3). Chronic viral infection provides a molecular basis for tumorigenesis by directly modulating host cell proliferation, inhibiting apoptosis, and inducing genomic instability (4, 5).

However, the impact of viruses on cancer development extends beyond direct cellular changes. Increasing evidence indicates that chronic viral infections significantly reshape the tumor immune microenvironment (TIME) through sustained immune stimulation and suppression (6). Persistent viral antigens drive CD8^+^ T cell exhaustion, impair natural killer (NK) cell function, and enable immune evasion, while promoting the accumulation of regulatory T cells (Tregs), Myeloid-Derived Suppressor Cells (MDSCs), and immunosuppressive cytokines (7). This creates an immune milieu conducive to tumor survival and progression. In addition, chronic viral infection can modulate immune cell infiltration and function through pro-inflammatory cytokines, exosomes, or microvesicle-mediated signaling, thereby facilitating tumor cell colonization and distant metastasis (8).

This mini-review focuses on how chronic viral infections shape the tumor microenvironment via immune regulatory mechanisms and drive tumor metastasis. We summarize viral effects on innate and adaptive immunity, dissect the molecular mechanisms of CD8^+^ (Cluster of Differentiation 8–positive) T lymphocyte cell exhaustion and immune evasion, and discuss the role of virus-driven immunosuppressive microenvironments in tumor metastasis. This analysis aims to provide insights into the immune pathogenesis of virus-associated cancers and support the development of novel therapeutic strategies targeting the virus–immune–metastasis axis.

## 2 Chronic viral infection shapes the tumor immune microenvironment

**Figure 1:**
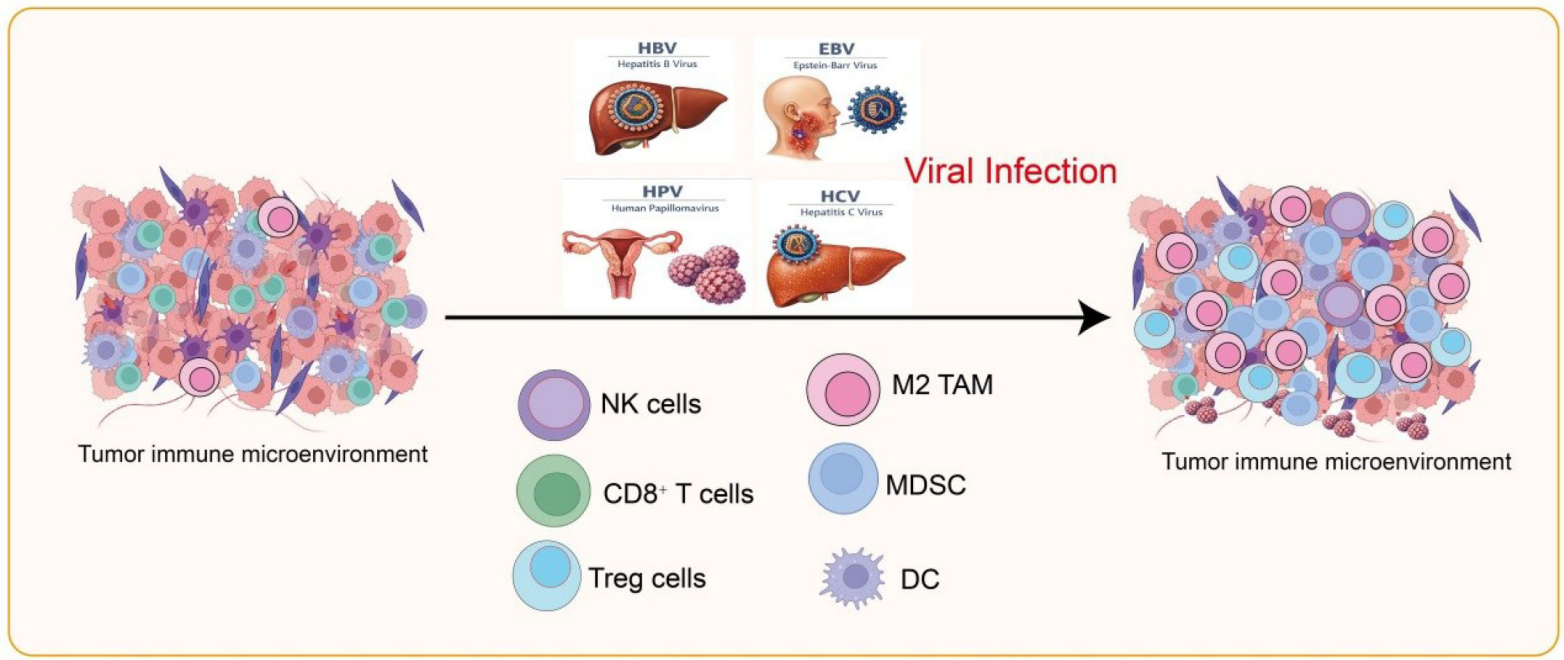
Impact of viral infections on the tumor immune microenvironment. Viral infections, including HBV, HCV, HPV, and EBV, modulate immune cell composition and function within the tumor microenvironment. NK cells, CD8^+^ T cells, and DCs show downregulated activity, whereas Treg cells, M2-TAMs, and MDSCs are functionally upregulated.

Chronic viral infections promote tumor development not only by directly altering host cell functions but also by reshaping the TIME through complex immunoregulatory mechanisms, creating conditions favorable for tumor growth and metastasis (9, 10). This remodeling primarily involves establishing an immunosuppressive environment, chronic inflammation with pro-tumor signaling, impaired antigen presentation and immune evasion, and regulation mediated by exosomes and microvesicles (11). HBV,HCV, HPV, and EBV are major oncogenic viruses that differ in genome type, target cells, transmission routes, and mechanisms of immune modulation, ultimately contributing to distinct virus-associated cancers (**Table 1****) (**12). As illustrated in **Figure 1**, these viral infections suppress the activity of cytotoxic NK and CD8^+^ T cells while enhancing immunosuppressive populations including regulatory Tregs, M2 tumor-associated macrophages, and myeloid-derived suppressor cells, thereby reshaping the immune landscape in favor of tumor survival and growth.

**Table 1:** Oncogenic viruses and tumor immune modulation.

Feature/virus	HBV	HCV	HPV	EBV
Virus Type	DNA virus, partially double-stranded	RNA virus, single-stranded positive-sense	DNA virus, double-stranded	DNA virus, double-stranded
Genome Size	~3.2 kb	~9.6 kb	~8 kb	~170 kb
Virus Family	Hepadnaviridae	Flaviviridae	Papillomaviridae	Herpesviridae
Replication Site	Nucleus (via reverse transcription)	Cytoplasm	Nucleus	Nucleus
Transmission	Blood, sexual, perinatal	Blood, needle sharing, transfusion	Sexual, skin-to-skin contact	Saliva, blood, organ transplant
Target Cells	Hepatocytes	Hepatocytes	Epithelial cells (cervix, anogenital)	B cells, epithelial cells
Oncogenic Mechanism	Integration into host genome, chronic inflammation	Chronic inflammation, oxidative stress	E6/E7 proteins inactivate p53/Rb	Latent infection, immortalization of B cells, LMP1/EBNA proteins
Associated Cancers	HCC	HCC	Cervical cancer, anogenital cancers, oropharyngeal cancer	Burkitt lymphoma, Hodgkin lymphoma, nasopharyngeal carcinoma
Immune Cell Changes	Chronic infection: exhausted CD8+ T cells, impaired NK cells	Chronic infection: exhausted CD8+ T cells, altered NK cells, increased Tregs	Local immune evasion: reduced antigen presentation, altered Langerhans cells	Altered B cell activation, T cell exhaustion, immune evasion via latent proteins
Chronic Infection Risk	High (~5–10% of adults)	Moderate (~50–85% of infections become chronic)	Low (most cleared, persistent infection with high-risk types)	Low for symptomatic disease, lifelong latent infection common
Vaccine Availability	Yes (effective)	No (treatment available)	Yes (HPV vaccine)	No

HBV, Hepatitis B virus; HCV, Hepatitis C virus; HPV, Human papillomavirus; EBV, Epstein–Barr virus; HCC, Hepatocellular carcinoma; Tregs, Regulatory T cells, NK cells, Natural killer cells; DNA, Deoxyribonucleic acid; RNA, Ribonucleic acid; LMP1, Latent membrane protein 1; EBNA, Epstein–Barr nuclear antigen.

### 2.1 Establishment of an immunosuppressive environment

Chronic viral infection significantly promotes the accumulation and activity of immunosuppressive cells, generating “cold” tumor microenvironments (13). Tregs and MDSCs are markedly increased in various virus-associated tumors. For instance, Tregs are elevated in HBV-related hepatocellular carcinoma, suppressing CD8^+^ T cell and NK cell cytotoxicity (14). Viral infection also induces anti-inflammatory cytokines, such as IL-10 and TGF-β, which further enhance immunosuppression by inhibiting effector T cell function and promoting the expansion of suppressive cells (15). Concurrently, CD8^+^ T cells display an exhausted phenotype, characterized by high expression of Programmed Cell Death Protein 1 (PD-1), T-cell Immunoglobulin and Mucin-Domain Containing Protein 3 (TIM-3), and Lymphocyte-Activation Gene 3 (LAG-3), while NK cell activity declines, leading to impaired antitumor immunity and facilitating immune escape (16).

### 2.2 Chronic inflammation and pro-tumor signaling

Chronic viral infection often induces persistent local or systemic low-grade inflammation. This prolonged inflammatory state not only causes DNA damage and increases mutation risk but also supports tumor proliferation through pro-growth factors such as vascular endothelial growth factor (VEGF), epidermal growth factor (EGF), and hepatocyte growth factor (HGF) (17). Key inflammatory signaling pathways, including nuclear factor kappa-light-chain-enhancer of activated B cells (NF-κB) and signal transducer and activator of transcription 3 (STAT3), are continuously activated in chronic infection-associated tumors, driving both pro-tumor and immunoregulatory gene expression (18). Tumor-associated inflammatory cells, such as tumor-associated macrophages (TAMs) and neutrophils, secrete chemokines including C-C motif chemokine ligand 2 (CCL2) and C-X-C motif chemokine ligand 8 (CXCL8/IL-8), which recruit immunosuppressive immune populations—such as myeloid-derived suppressor cells (MDSCs) and regulatory T cells (Tregs)—into the tumor, thereby promoting immune evasion (19). For example, Interleukin-21 (IL-21) can enhance MDSC-mediated immunosuppression, reducing liver inflammation in chronic HBV infection, suggesting that the IL-21–MDSC axis may regulate hepatic immune responses and optimize anti-inflammatory therapy (20).

### 2.3 Antigen presentation and immune evasion

Viral infections interfere with antigen presentation and immune recognition through multiple mechanisms. Chronic viral or tumor cells often downregulate MHC I/II expression, impairing antigen processing and presentation, and reducing CD8^+^ T cell recognition and cytotoxicity (21). For instance, EBV LMP1 disrupts antigen-processing pathways and decreases surface antigen presentation in tumor cells (22). Viruses also induce inhibitory receptors and cytokines and modulate immune checkpoint signaling, establishing immune evasion pathways that protect tumor cells from host immunity (23). Antigen-presenting cells (APCs), including macrophages, dendritic cells (DCs), Langerhans cells (LCs), and B cells, are the primary effector cells in peripheral immune microenvironments (24). EBV infects autoreactive antinuclear antigen B cells in systemic lupus erythematosus (SLE), reprogramming them into activated antigen-presenting cells that drive T peripheral helper cell activation and systemic autoimmunity. This mechanistic link provides direct evidence that EBV can promote SLE by expanding and functionally altering nuclear antigen–reactive B cells (24). In peripheral immune microenvironments, macrophages polarize into a proinflammatory M1 phenotype under VEGF-, IL-6-, and IL-8, rich conditions, supporting CD4^+^ T cell infiltration (25). Extending these principles to virus-associated tumor microenvironments, during cervical lesion progression, macrophages shift to an immunosuppressive M2 phenotype, inhibiting CD8^+^ T cell function and recruiting regulatory Tregs and MDSCs (26). In high-grade serous ovarian cancer (HGSOC) tumor cells, B7-H3 (an immune checkpoint protein with immunosuppressive functions) expression promotes tumor immune evasion via the CCL2-CCR2–M2 macrophage axis, reducing IFNγ^+^CD8^+^ T cell infiltration. Targeting B7-H3 or the CCL2-CCR2 pathway may therefore represent a promising strategy to overcome immune suppression in PD-L1–low, nonimmunoreactive HGSOC (26). Similarly, high-risk HPV reduces chemokine and E-cadherin expression in keratinocytes, limiting Langerhans cell (LC) migration to infection sites and impairing LC and DC retention within the infected epidermis, thereby weakening local antiviral responses (27).

### 2.4 Exosome- and microvesicle-mediated immune regulation

Exosomes and microvesicles secreted by virus-infected cells play a critical role in TIME modulation (28). These vesicles can carry viral proteins, miRNAs, or immunoregulatory molecules that directly suppress CD8^+^ T cell and NK cell activity. For example, exosomes from HBV-infected hepatocytes carrying HBx protein and miR-21 inhibit T cell proliferation and promote MDSC recruitment (29). For example, CircCCAR1 promotes HCC growth and metastasis through a circCCAR1/miR-127-5p/WTAP feedback loop and enhances PD-L1 transcription via CCAR1–β-catenin interaction. Exosomal circCCAR1 induces CD8^+^ T cell dysfunction and resistance to anti-PD-1 therapy, representing a potential target to overcome immunosuppression in HCC (29). Beyond remodeling the local tumor environment, exosomes can form pre-metastatic niches in distant tissues, altering immune cell composition and function to facilitate tumor colonization and metastasis (30). Notably, plant-derived GELN exosomes carrying osa-miR164d can reprogram macrophage polarization by targeting TAB1 and downregulating NF-κB, suppressing intestinal inflammation (31). Based on this concept, engineered biomimetic exosomes (osa-miR164d-MGELNs) effectively reprogram macrophages and alleviate colitis symptoms, representing an innovative cross-species miRNA delivery strategy (31). Similarly, circPRKD3 in glioma stem cell (GSC) exosomes binds HNRNPC in an m6A-dependent manner, accelerates IL6ST mRNA degradation, and inhibits STAT3 signaling, reprogramming tumor-associated macrophages to secrete CXCL10 and recruit CD8^+^ T cells against glioblastoma (32). Brain-targeted lipid nanoparticles delivering circPRKD3 combined with immune checkpoint blockade show strong antitumor synergy, highlighting a promising RNA-based immunotherapeutic approach for GBM (32).

## 3 Immune mechanisms of chronic viral infection–driven tumor metastasis

Chronic viral infections not only promote local tumor growth but also facilitate distant metastasis through complex immune regulatory mechanisms (33). By altering antitumor immunity and remodeling both the tumor microenvironment and distant tissue microenvironments, viruses enable tumor cells to evade immune surveillance and successfully colonize distant organs.

### 3.1 Immune escape facilitates metastasis

CD8^+^ T cells and NK cells are central effectors of antitumor immunity. In virus-associated tumors, these effector cells often display exhaustion or functional impairment. Persistent antigen stimulation and immunosuppressive factors produced by oncogenic viruses (e.g., HBV, HCV, HPV, EBV), such as viral PD-L1, IL-10, and TGF-β, reduce cytokine secretion and proliferation of CD8^+^ T cells while decreasing NK cell cytotoxicity (34). This immune escape allows circulating or distant tumor cells to evade host surveillance, increasing metastatic potential (34). For instance, CD8^+^ T cell exhaustion in HBV-related hepatocellular carcinoma correlates closely with intravascular tumor dissemination (35). Compared with uninfected samples, HPV-infected and cervical cancer tissues show significant increases in myeloid and NK/T cell populations, while epithelial cell proportions decrease (36). Functional enrichment analysis reveals activation of αβ T cells, T cell receptor signaling, and neutrophil chemotaxis, highlighting substantial immune remodeling during HPV-driven tumor progression. SPP1^+^ macrophages interact with tumor cells and exhausted T cells via the SPP1–CD44 axis, shaping an immunosuppressive microenvironment (37). Moreover, HPV16-driven cervical cancer releases IL-1 family cytokines that skew bone marrow myelopoiesis toward immunosuppressive neutrophils, dampening systemic T cell immunity. Blocking IL-1 signaling (e.g., anti-IL1RAP) inhibits this neutrophil expansion, restoring the efficacy of otherwise ineffective HPV16 E7 vaccines, which can be further enhanced by combining with anti- Cytotoxic T-Lymphocyte–Associated Protein 4 (CTLA-4) therapy (38).

### 3.2 Immune cell–mediated metastatic microenvironment

Chronic viral infection indirectly promotes metastasis by regulating immune cell populations. MDSCs and tumor-associated macrophages (TAMs) are expanded in virus-associated tumors and secrete pro-migratory factors, including VEGF, matrix metalloproteinases (MMPs), and chemokines (39). VEGF supports tumor angiogenesis, providing channels for tumor cells to enter circulation, while MMPs degrade the extracellular matrix, facilitating invasion and migration (40). TAMs also secrete chemokines such as CCL2 and CCL5, recruiting immune cells to the metastatic microenvironment and further enhancing tumor invasiveness (41). For example, MARCO is a key regulator of MDSC differentiation and immunosuppression in breast cancer (42). Genetic deletion or antibody-mediated inhibition of MARCO suppresses tumor growth, reduces immunosuppressive MDSCs and TAMs, and enhances CD8^+^ T cell and NK cell infiltration (42). Combining MARCO inhibition with PD-1 blockade produces synergistic antitumor effects, highlighting MARCO as a promising immunotherapy target.

### 3.3 Chronic inflammation and angiogenesis

Persistent low-grade inflammation induced by chronic viral infection maintains a pro-tumor microenvironment and promotes metastasis via angiogenesis (43). Continuous inflammatory signaling activates NF-κB and STAT3, inducing VEGF, IL-6, and other pro-angiogenic factors that drive new vessel formation. These vessels supply nutrients and oxygen while providing physical routes for tumor cells to enter circulation (43). HPV16 E6 upregulates hypoxia-inducible factor 1α (HIF-1α) and promotes VEGF expression, strongly enhancing angiogenesis (44). MMPs, particularly MMP-2 and MMP-9, degrade the vascular basement membrane, exposing VEGF-A receptors on endothelial cells and stimulating further angiogenesis (45). Tumor cells exploit these vascular gaps to intravasate, facilitating distant metastasis. HPV16 E6/E7 promotes HIF-1α and GLUT1 expression in lung cancer cells by downregulating the tumor suppressor RRAD, which activates NF-κB via nuclear translocation of p65. This HPV-RRAD-p65-HIF-1α-GLUT1 axis highlights RRAD as a critical regulator in HPV-related lung cancer and a potential therapeutic target (46).

### 3.4 Exosomes and cytokines establish pre-metastatic niches

Chronic viral infection also establishes pre-metastatic niches in distant tissues via exosomes and cytokines (47). Tumor and virus-infected cells release exosomes carrying viral proteins, miRNAs, or immunosuppressive factors, reshaping the immune landscape of distant organs (47). Exosomes can recruit MDSCs or inhibit NK cell activity, providing immune protection for circulating tumor cells to colonize and proliferate (48). Inflammatory mediators such as TNF-α and IL-6 can act remotely to modulate stromal and endothelial cell functions, optimizing the metastatic “landing” environment (48). For example, during chronic viral infections, a subset of virus-specific CD8^+^ T cells expressing Tcf1 sustains T cell responses despite terminal exhaustion, displaying memory-like features and inhibitory receptors such as PD-1 and Lag-3 (49). This population represents a key target for interventions aimed at enhancing antiviral immunity and may be influenced by viral-derived factors, including exosomes (49). In chronic viral infections, PD-1+ Tcf1+ stem-like CD8^+^ T cells are not irreversibly exhausted and can be reprogrammed by PD-1 blockade combined with IL-2 to generate highly functional effector CD8^+^ T cells (50). This synergy relies on IL-2 signaling through the high-affinity CD25 receptor and highlights a potential strategy for enhancing antiviral immunity and informing cancer immunotherapy (50).

## 4 Chronic viral infection–associated signaling pathways: NF-κB, STAT3, PD-1/PD-L1, and TGF-β

**Figure 2:**
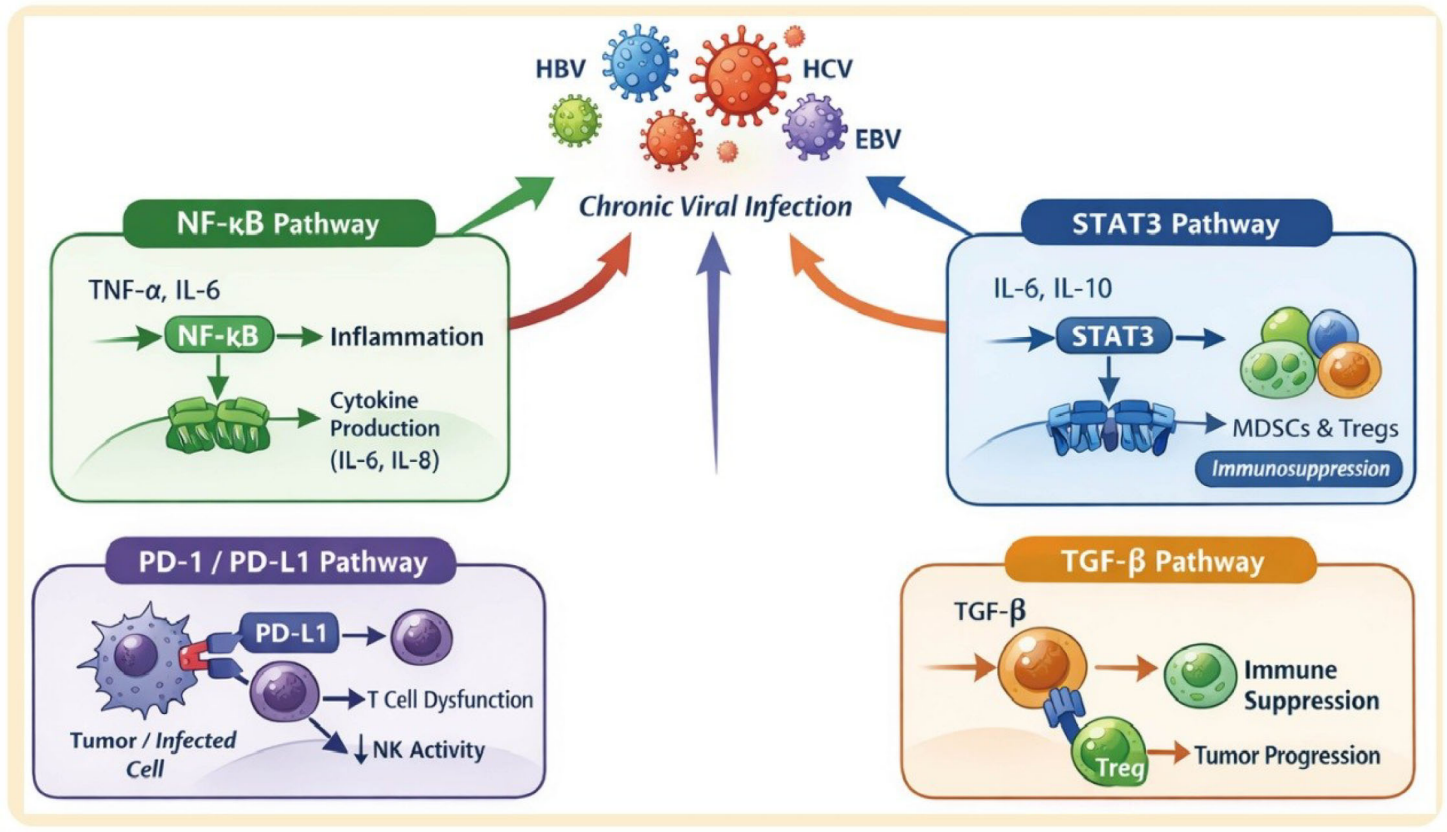
Chronic viral infection–associated signaling pathways modulating immune responses. Schematic illustration showing the key signaling pathways activated during chronic infections with oncogenic viruses such as HBV, HCV, HPV, and EBV. TNF-α and IL-6 activate the NF-κB pathway, promoting inflammation and cytokine (IL-6, IL-8) production. The STAT3 pathway, activated by IL-6 and IL-10, drives immunosuppression via the expansion of MDSCs and Tregs. PD-1/PD-L1 interactions between infected or tumor cells and T cells induce T cell dysfunction and reduce NK cell activity. TGF-β signaling promotes immune suppression and tumor progression through Treg activation.

Chronic viral infections regulate the tumor immune microenvironment through multiple signaling pathways, promoting tumor growth and metastasis. Key pathways include NF-κB, STAT3, PD-1/PD-L1, and TGF-β. Specific viral proteins can directly modulate these pathways, enhancing pro-tumor and immunosuppressive signaling (**Figure 2**).

### 4.1 NF-κB: chronic inflammation and pro-metastatic factors

The NF-κB pathway is persistently activated in chronic virus-associated tumors and serves as a central regulator of chronic inflammation and pro-metastatic factor expression (51). Chronic infections, including HBV, HCV, and EBV, activate IκB kinase (IKK), maintaining NF-κB activity. Activated NF-κB upregulates pro-inflammatory cytokines (e.g., TNF-α, IL-6), chemokines (e.g., CCL2, CXCL8), and pro-angiogenic factors such as VEGF (51). This sustains chronic inflammation, enhances MDSC and TAM activity, and promotes tumor invasion and metastasis. For example, EBV LMP1 mimics CD40 signaling to continuously activate NF-κB, driving expression of pro-inflammatory and pro-migratory genes (52).

### 4.2 STAT3: regulator of immunosuppressive cells and metastasis

STAT3 is another key node persistently activated in virus-associated tumors, linking immunosuppression and metastasis (53). Chronic viral infections activate STAT3 through pro-inflammatory cytokines such as IL-6 and IL-10, promoting Treg and MDSC proliferation and function while suppressing CD8^+^ T cell activity (53). STAT3 activation also enhances VEGF and MMP expression, facilitating angiogenesis and extracellular matrix degradation, thereby supporting metastasis (54). HBV HBx protein further promotes STAT3 activation via PI3K/Akt and JAK/STAT3 pathways, driving hepatocellular carcinoma progression.

### 4.3 PD-1/PD-L1: central mechanism of immune escape

The PD-1/PD-L1 immune checkpoint is a key mechanism of immune escape in virus-associated tumors (55). Chronic viral infection induces high PD-1 expression on CD8^+^ T cells, while tumor or infected cells upregulate PD-L1, inhibiting T cell proliferation and cytokine production through PD-1/PD-L1 binding (55). Persistent activation of this pathway weakens local antitumor immunity and allows circulating tumor cells to evade immune surveillance in distant organs, facilitating metastasis. In EBV-associated tumors, LMP1 upregulates PD-L1, directly contributing to immune evasion (56).

### 4.4 TGF-β: immunosuppression and epithelial–mesenchymal transition

TGF-β is a critical mediator of immunosuppression and metastasis in chronic virus-associated tumors (57). TGF-β promotes Treg expansion, suppresses effector T cell and NK cell function, and establishes an immunosuppressive microenvironment (58). Simultaneously, TGF-β drives EMT, enhancing tumor cell migration and invasiveness. Viral proteins such as HBV HBx and EBV LMP1 upregulate TGF-β expression or enhance downstream signaling, further promoting immune escape and metastasis (59). In pediatric EBV infection, macrophage PD-L1 expression is regulated by viral antigens, with CD163^+^ M2 macrophages correlating with latent LMP1 in primary infection and CD68^+^ M1 macrophages correlating with lytic BMRF1 in reactivation. These results suggest that EBV modulates macrophage-mediated immune exhaustion early in infection, highlighting potential targets for EBV-associated tumor therapy (60).

## 5 Potential therapeutic strategies and research prospects

Recent studies have proposed several intervention strategies targeting the immunosuppressive and pro-metastatic features of chronic virus-associated tumors. These approaches aim to restore antitumor immunity, block virus-mediated pro-metastatic pathways, and improve clinical outcomes. Immune checkpoint inhibitors (ICIs), such as PD-1/PD-L1 and CTLA-4 blockers, have demonstrated efficacy in various virus-associated cancers (61). For example, patients with EBV-positive nasopharyngeal carcinoma or HBV-related hepatocellular carcinoma often show higher response rates to PD-1 inhibitors than those with non-virus-associated tumors, likely due to virus-driven PD-L1 overexpression and unique immune microenvironment characteristics (62). ICIs can partially restore CD8^+^ T cell and NK cell activity, reverse immunosuppression, and inhibit tumor progression and metastasis. Viral clearance or suppression can reduce persistent antigen stimulation, alleviate CD8^+^ T cell exhaustion, and improve the immune microenvironment (63). For instance, combining antiviral therapy against HBV or HCV with immunotherapy can lower viral replication while enhancing antitumor immunity, providing a novel approach for liver cancer prevention and treatment. Such combination strategies may also reduce the risk of distant metastasis (64).

Exosomes play a pivotal role in immune regulation and pre-metastatic niche formation in virus-associated tumors. Targeting or inhibiting exosome secretion, transport, or function could serve as a novel immunotherapeutic approach (65). Inhibiting exosome release from tumor or virus-infected cells may reduce the accumulation of immunosuppressive miRNAs or proteins in the microenvironment, restore CD8^+^ T cell and NK cell activity, and suppress metastasis. Changes in the immune microenvironment of virus-associated tumors can also serve as biomarkers for early diagnosis, therapeutic response monitoring, and prognostic prediction (66). Indicators such as serum exosomal miRNAs, proportions of circulating immunosuppressive cells, and PD-L1 expression levels can dynamically reflect immune microenvironment status and guide personalized immunotherapy. Future development of high-throughput, multi-modal biomarker profiling will facilitate precise assessment of the immune status and metastatic risk in virus-associated cancers.

## 6 Conclusion

Chronic viral infections play a critical role in tumor initiation, immune evasion, and distant metastasis through multi-layered immunoregulatory mechanisms. Key processes include the accumulation of immunosuppressive cells, CD8^+^ T cell exhaustion, chronic inflammation, angiogenesis, and exosome-mediated local and distant immune modulation (67). Viral proteins, such as EBV LMP1 and HBV HBx, further enhance immunosuppression and pro-metastatic signaling by modulating key pathways including NF-κB, STAT3, PD-1/PD-L1, and TGF-β. Future research should focus on elucidating the molecular and cellular mechanisms of the virus–immune–metastasis axis, including key factors driving pre-metastatic niche formation. Comprehensive analysis of exosomes, immune cells, and cytokines in distant tissues will provide insights for metastasis intervention. Developing novel therapies that combine antiviral and immunotherapeutic strategies or target exosomes and immunosuppressive pathways may restore antitumor immunity and prevent metastasis. By integrating mechanistic studies with clinical translation, these approaches have the potential to provide more precise immunotherapeutic strategies for virus-associated tumors, reduce metastasis incidence, and improve patient prognosis.
